# Blood glucose management in hospitalized patients: a review of current literature

**DOI:** 10.1097/MS9.0000000000002991

**Published:** 2025-02-26

**Authors:** Siddharth Patel, Amogh Reddy, Mc Anto Antony, Mrudula Thiriveedi, Prutha Pathak, Sujatha Baddam, Hinal Rathi

**Affiliations:** a Decatur Morgan Hospital, Decatur, Alabama, USA; b Alabama College of Osteopathic Medicine, Dothan, Alabama, USA; c Medical University of South Carolina/AnMed campus, Anderson, South Carolina, USA; d North Alabama Medical Center, Florence, Alabama, USA; e Huntsville Hospital, Huntsville, Alabama, USA; f Internal Medicine Resident, University of Alabama at Huntsville, Huntsville, Alabama, USA

**Keywords:** blood glucose control, diabetes management, hospitalized patients, hyperglycemia

## Abstract

Diabetes mellitus is a chronic medical condition which affects millions of adults worldwide. It can result in various complications and is associated with a higher rate of hospitalizations. Blood glucose management in hospitalized patients is a critical aspect of care, which is important for preventing complications, improving patient outcomes, and reducing the length of hospital stay. Blood glucose control is difficult to achieve secondary to multiple factors involved in its regulation (e.g. type of medical illness, corticosteroid use, and enteral feeding) as well as varying evidence to determine different aspects of it. Our goal is to summarize the existing evidence from observational studies, clinical trials, and various society guidelines on blood glucose management in the hospitalized setting.

## Introduction

Diabetes mellitus (DM) is one of the most common metabolic disorders which affects approximately 530 million adults worldwide^[1,2]^. The prevalence of type 2 DM in the US is around 8.5–11.3%^[3–5]^. There were 16 million emergency department (ED) visits and 7.8 million hospital stays for people with diabetes in the US in 2016^[6]^. Patients with diabetes have a 3-4-fold higher rate of hospitalization than those without it^[7,8]^. Moreover, they have a higher length of stay as compared to non-diabetics^[9,10]^. Thirty percent of diabetics require rehospitalization in any given year^[11–13]^.

**HIGHLIGHTS**

- Blood glucose management in hospitalized patients is critical for preventing complications, improving patient outcomes, and reducing the length of hospital stay.
- There is varying evidence to determine the target blood glucose level.
- We summarize the existing evidence from observational studies, clinical trials, and various society guidelines on blood glucose management in the hospitalized setting.
- Majority of societies recommend modest blood glucose control with a blood glucose target of <180 mg/dL while avoiding hypoglycemia.

Hyperglycemia (blood glucose >140 mg /dL) is reported in 22-46% of non-critical hospitalized individuals. It is associated with higher risk of complications and mortality^[14,15]^. Although, the need for inpatient blood glucose control is well-recognized, the evidence has often been inadequate to determine various aspects of it, e.g. blood glucose control in critically ill vs not critically ill, surgical vs non-surgical patients, avoiding harms associated with hypoglycemia, etc. Many medical societies, therefore, have discrepant recommendations on target blood glucose levels in hospitalized individuals. In this narrative review, we summarize the evidence from observational studies, clinical trials, and various society guidelines on blood glucose management in hospitalized individuals.

### Definition and epidemiology

Hyperglycemia is defined as a blood glucose concentration of greater than 140 mg/dL (7.8 mmol/L)^[15,16]^. It occurs in hospitalized patients with known diabetes, undiagnosed diabetes, and as “stress hyperglycemia” due to acute illness which resolves at the time of discharge^[15]^. The American Association of Clinical Endocrinologists (AACE) consensus on inpatient hyperglycemia defined stress hyperglycemia or hospital-related hyperglycemia as any blood glucose concentration >140 mg/dL (>7.8 mmol/L) in patients without a prior history of diabetes^[15,16]^.

Observational studies have found high rates of hyperglycemia in hospitalized patients including 22% to 46% of non-critically ill patients, 41% of critically ill patients with acute coronary syndromes, 44% of heart failure patients, and 80% of patients after cardiac surgery^[8,15–18]^. Furthermore, a 2017 report, based on point-of-care bedside glucose tests, gathered data from 3.5 million people (575 hospitals), including 635,359 ICU and 2,831,436 non-ICU patients found that 32.2% of ICU patients and 32.0% of non-ICU patients had hyperglycemia (>180 mg/dL)^[19]^.

### Harms of hyperglycemia in hospitalized patients

Hyperglycemia can cause glycosuria, osmotic diuresis, electrolyte abnormalities, and hypovolemia. Additionally, severe hyperglycemia can cause neutrophil dysfunction and increase the susceptibility to infections^[20–22]^. It can worsen neuronal injury by tissue acidosis and disruption of the blood-brain barrier in patients with acute stroke, which in turn can increase the size of brain infarct and worsen the outcomes^[23,24]^. Diabetic individuals also have a significantly higher risk of recurrent cardiovascular events and mortality after an acute myocardial infarction^[25]^. They have a higher risk of cardiac arrhythmia, heart failure, renal failure, and cardiogenic shock as compared to non-diabetics^[26–29]^. In addition, inpatient hyperglycemia is associated with an increased risk of mortality, an increased length of hospital stay, a higher admission rate to the critical care unit, and a higher rate of need for nursing home care after discharge^[14,30]^. On the other hand, attempting strict glycemic control is associated with an increased risk of hypoglycemia, morbidity, and mortality in hospitalized individuals^[19,31–33]^. Hence, maintaining stable glycemic balance, preventing adverse glycemic events, and ensuring a smooth transition to appropriate outpatient care are important.

### Barriers to optimum diabetes control in hospitalized patients

Although the beneficial effects of optimum blood glucose control in hospitalized individuals are well-recognized, it is not always easy to achieve. Clinical conditions such as pancreatitis, use of glucocorticoids, and parenteral nutrition can contribute to hyperglycemia. Change in medication administration timing because of hospital logistics, prolonged reliance on the use of sliding scale insulin, variable timing of meal intake, different diet than home, and use of enteral tube feeding are other factors responsible for altered blood glucose control in hospitalized patients. Studies also identified omission of discussions during rounds and unpreparedness to address blood glucose control during rounds as the barriers to achieve optimum blood glucose control^[16]^. Poor communication between healthcare team members, lack of standardized insulin protocols and inconsistent dose adjustment, inadequate access to bedside glucose monitoring, and lack of knowledge among physicians on blood glucose control in hospitalized patients are additional factors responsible for hyperglycemia. The unavailability of bedside blood glucose monitors, adequate number of nurses, and endocrinological expertise further make management of hyperglycemia challenging inpatient. Conversely, decreased appetite associated with any illness and the need for staying nothing by mouth before procedures can contribute toward hypoglycemia. Abnormal liver and kidney function can impair metabolism of insulin and oral hypoglycemic agents and can contribute to hypoglycemic events as well. Since the net effect of the above-mentioned counteracting factors is difficult to predict and severe hypoglycemia can lead to significant comorbidities including seizure, coma, and death^[17]^, the target blood glucose levels are generally set higher in hospitalized patients than in non-hospitalized patients.

## Methods

### Literature review

We performed a thorough review of the literature to gather the latest guidelines on inpatient hyperglycemia management from various medical professional associations, reviews, and clinical trials. We used PubMed and Google Scholar. Keywords included “hyperglycemia,” “hospitalized patients,” “diabetes,” “critically ill patients,” “non critically ill patients,” “surgical patients,” “pharmacotherapy,” and “glycemic goals.” We then selected organizations that have published guidelines for hyperglycemia management in non-critically ill patients, surgery patients, critically ill patients, and terminally ill patients. To review the literature in a succinct but efficient manner, we prioritized data collection and review of the most prominent literature. Since the institutional guidelines have a wide variability in the recommendations, we decided to choose the organizational guidelines which are widely followed and regarded as standard. We focused on four organizations including the American Diabetes Association (ADA), Society of Critical Care Medicine (SCCM), American College of Physicians (ACP), and the Society of Thoracic Surgeons (STS). The most recently updated guidelines from these organizations were considered. Two authors independently reviewed the guidelines for hyperglycemia management in the hospital setting in detail and compiled the information into a word document.

## Results

**Figure 1.**
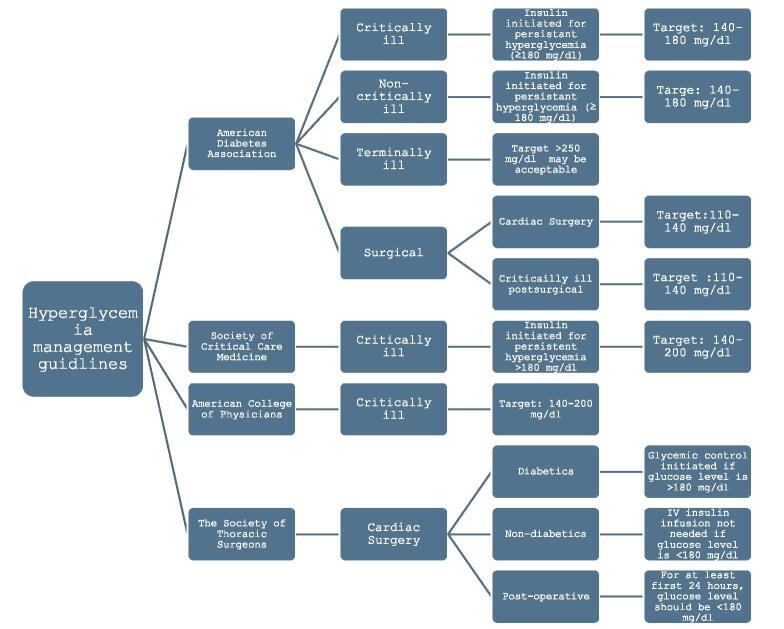
Overview of target blood glucose level in hospitalized patients according to various society guidelines.

The results from the literature review are summarized in a flow diagram (Fig. 1).

### ADA guidelines

The ADA recommends inpatient glycemic targets based on the findings of a trial conducted by Van den Berghe *et al* and the NICE-SUGAR trial^[33]^. Van den Berghe *et al* showed a reduction in mortality by 40% in surgical intensive care unit patients having a stricter target glucose of 80–110 mg/dL (4.4–6.1 mmol/L) compared with a more lenient target glucose of 180–215 mg/dL (10–12 mmol/L)^[33]^. However, a follow up-study in critically ill hospitalized patients, the Normoglycemia in Intensive Care Evaluation and Survival Using Glucose Algorithm Regulation (NICE-SUGAR) trial, compared critically ill patients randomized to intensive glycemic management (80–110 mg/dL) to a group with more moderate glycemic targets (140–180 mg/dL [7.8–10.0 mmol/L]). The NICE-SUGAR trial showed that there was a slight but higher rate of mortality in the intensive glycemic management group^[33]^ along with 10- to 15-fold greater rates of hypoglycemia. These findings are supported by several meta-analysis studies and randomized controlled trials^[33]^. Although both studies target critically ill patients, the results have been extended to hospitalized patients without critical illness as well^[33]^.

### SCCM guidelines

The SCCM recommends that the clinicians should initiate glycemic management protocols and procedures to treat persistent hyperglycemia ≥ 10 mmol/L (180 mg/dL) in critically ill adults. Their multi-disciplinary panel considers it to be good practice to manage persistent hyperglycemia with evaluation of glucose intake, additional monitoring, and insulin therapy^[34]^.

### ACP guidelines

The ACP states that intensive insulin therapy (IIT) with a goal of achieving normal or near-normal blood glucose in patients with or without diabetes does not provide substantial benefits and may lead to harm^[35]^. They also suggest that utilizing IIT for maintaining strict glucose control compared to a more lenient glucose control neither garners any reduction in mortality or length of hospital stay. In fact, the ACP cautions that IIT may increase the risk for severe hypoglycemia, especially in critically ill patients^[35]^.

### STS guidelines

The STS recommends maintaining blood glucose levels below 180 mg/dL during cardiac surgery citing reduced mortality, reduced morbidity, lower incidence of wound infections, reduced hospital length of stay, and enhanced long-term survival^[36]^.

## Discussion

The blood glucose level is determined by dietary intake, endogenous glucose production via gluconeogenesis, or glycogenolysis and its utilization by peripheral tissues^[37]^. Measurement of HbA1C is indicated in people with hyperglycemia without a history of diabetes to differentiate between stress induced hyperglycemia and previously undiagnosed diabetes^[36–39]^. The Endocrine Society and the UK Joint British Diabetes Societies for Inpatient Care recommendations indicate that people hospitalized with both hyperglycemia >140 mg/dL (7.8 mmol/L) and an HbA1C of 6.5% (48 mmol/mol) or higher can be identified as having diabetes^[15,36]^.

Uncontrolled hyperglycemia in hospitalized patients with or without a previous diagnosis of diabetes is associated with adverse outcomes and longer length of stay^[38]^. However, avoiding hypoglycemic events is equally important. This can get complex especially in patients with acute illness, inconsistent caloric intake, and changes in home medications^[38]^. Insulin is the preferred agent in hospitalized patients because of its reliable action and ease of dose titration^[38]^. It can be provided either as a basal/bolus or on a correction scale insulin regimen^[38]^. A study comparing scheduled basal/bolus insulin to correction scale insulin showed a significantly higher percentage of patients achieving goal glucose levels in the basal/bolus (66 vs 38%) without an increase in hypoglycemia^[38]^.

Oral hypoglycemics use is not recommended in hospitalized individuals due to the risk of adverse effects as well as inadequate data on their safety and efficacy in this setting (Table 1) ^[38,39]^, although the EMBODY trial showed that acute myocardial infarction patients in the empagliflozin group had improvement in the heart rate as compared to the placebo^[40]^. SOLIST-WHF and EMPULSE trials on hospitalized heart failure patients showed beneficial effects of sodium-glucose cotransporter 2 (SGLT2) inhibitors on improving survival and quality of life and reduced exacerbation of heart failure events^[40]^. However, more studies are needed to establish the safety of SGLT2 inhibitors among hospitalized patients with severe illness, ketosis, during prolonged fasting and perioperatively^[41]^. Liraglutide, a glucagon-like peptide 1 (GLP1) agonist has been shown to provide effective glycemic control^[42]^. Short-acting exenatide twice daily plus basal insulin has improved glycemic control compared to basal insulin or exenatide alone^[42]^. However, larger randomized controlled trials are lacking to assess efficacy and safety of use of GLP1 agonists in the inpatient setting for hyperglycemia management^[42]^.

**Table 1:** Oral and injectable medications for the treatment of type 2 diabetes mellitus and risks associated with their use in hospitalized patients

Medication class	Examples	Risks and/or contraindications
Biguanides	Metformin	Patients requiring iodinated contrast studies, patients with renal insufficiency, severe liver disease, and congestive heart failure
Sulfonylureas	Glipizide, glimepiride, glyburide	Increased risk for hypoglycemia in patients with malnutrition and severe renal and liver disease
Meglitinides	Repaglinide, nateglinide	Increased risk for hypoglycemic episodes in patients not having adequate nutrition
Thiazolidinediones	Pioglitazone, rosiglitazone	Fluid retention
GLP-1 agonists	Tirzepatide, semaglutide, dulaglutide, liraglutide	Nausea, should be withheld in acutely ill patients
Amylin agonists	Pramlintide	Nausea, should be withheld in acutely ill patients
Alpha glucosidase inhibitors	Acarbose, miglitol	Contraindicated in patients with GI diseases (inflammatory bowel disease, partial bowel obstruction), and severe renal or hepatic disease)
SGLT-2 inhibitors	Canagliflozin, dapagliflozin, empagliflozin	Increased risk for urinary tract infections

The use of technology, e.g. continuous glucose monitoring (CGM), may also be utilized in the future to aid in blood glucose management in hospitalized patients. The real-time glucose trend obtained through CGM could help physicians make proactive and timely clinical decisions. CGM systems can decrease hypoglycemia and reduce hyperglycemia in hospitalized patients with diabetes. They are safe and accurate and can decrease the need for frequent fingerstick checks. This in turn can decrease resource utilization^[43]^.

While optimal blood glucose control is desirable, strategies to improve hypoglycemia also need to be strictly tailored to individual’s needs. A 2024 article published in the Diabetes journal states that an individual’s risk for hypoglycemia should be identified by their hypoglycemic history and risk factors including but not limited to recent hypoglycemic events, intensive insulin therapy, impaired hypoglycemic awareness, ESRD, cognitive impairment, or dementia^[44]^. Patient education by a trained diabetes educator on identifying hypoglycemia triggers, e.g. fasting, delayed meals, physical activity, and illness, has been emphasized in hypoglycemia management^[44]^.

## Conclusion

Hyperglycemia is associated with an increased risk of morbidity and mortality in hospitalized patients. On the other hand, strict glycemic control is also associated with poor outcomes. Most professional societies recommend target blood glucose between 140 and 180 mg/dL in the ICU and non-ICU settings; however, more stringent targets may be appropriate for post-surgical or cardiac patients. Ideally, glycemic targets should be individualized based on patient’s clinical status, co-morbidities, and risk for hypoglycemia. Physician education and close attention to blood glucose management during rounds are extremely important. Insulin is the preferred agent for blood glucose management in the hospital which can be provided as a basal/bolus dose or on a correction scale regimen. Oral hypoglycemics are not typically recommended, and more studies are needed to establish their safety and efficacy in the inpatient setting. To minimize resource utilization in achieving this utmost important goal in patient care, use of technology such as CGM is the future.

## Data Availability

Not applicable since this is a review article. None of the contents of the article is derived from any publicly available dataset.
